# Effectiveness and safety of tocilizumab combined with different high‐dose methylprednisolone regimens for acute necrotizing encephalopathy in children

**DOI:** 10.1002/ped4.70039

**Published:** 2026-01-27

**Authors:** Fei Li, Kechun Li, Chaonan Fan, Quan Wang, Suyun Qian

**Affiliations:** ^1^ Department of Pediatric Intensive Care Unit, Beijing Children's Hospital Capital Medical University, National Center for Children's Health Beijing China

**Keywords:** Acute necrotizing encephalopathy, Cytokine storm, Methylprednisolone, Neurological outcomes, Tocilizumab

## Abstract

**Importance:**

Acute necrotizing encephalopathy (ANE) is a rare but life‐threatening pediatric neurological disorder characterized by rapid progression and high mortality. Tocilizumab, an interleukin‐6 receptor antagonist, combined with high‐dose methylprednisolone [MP, ≥20 mg/(kg·day)], may improve outcomes, yet the optimal MP dosing strategy remains uncertain.

**Objective:**

To evaluate the comparative effectiveness and safety of two high‐dose MP regimens [20 mg/(kg·day) vs. 30 mg/(kg·day)], each in combination with tocilizumab for ANE.

**Methods:**

This retrospective cohort study included 23 ANE patients treated with tocilizumab and high‐dose MP at Beijing Children's Hospital from January 2023 to January 2025. Patients were divided into two groups based on the initial MP dosage: 20 mg/(kg·day) (*n* = 11) and 30 mg/(kg·day) (*n* = 12). Primary outcomes included mortality and anti‐inflammatory response. Secondary outcomes were the incidence of severe neurological sequelae, assessed using the pediatric overall performance category (POPC) score, and the frequency of treatment‐related adverse events.

**Results:**

Overall mortality rate was 26.1% with a lower rate observed in the 30 mg/(kg·day) group (16.7%) compared to the 20 mg/(kg·day) group (36.4%). Most patients (78.3%) had severe ANE (ANE Severity Score ≥5), and 91.3% presented with multi‐organ dysfunction and brainstem involvement. Both groups demonstrated significant reductions in procalcitonin, cerebrospinal fluid protein, and cerebrospinal cytokines after 3 days of MP therapy (*P* < 0.05). Compared with the 20 mg/(kg·day) group, the 30 mg/(kg·day) MP group had significantly lower rates of severe neurological sequelae (POPC score 4–6) at discharge (41.7% vs. 90.9%; *P* = 0.027) and at 6–12 months follow‐up (30.0% vs. 85.7%; *P* = 0.050). No statistically significant differences in adverse event rates were observed between the two groups (*P* > 0.05), and no tocilizumab‐related adverse events were reported.

**Interpretation:**

In pediatric ANE, tocilizumab combined with 30 mg/(kg·day) MP was associated with improved neurological outcomes compared with 20 mg/(kg·day), with comparable mortality and safety profiles. These findings suggest that a higher initial MP dose may offer neuroprotective advantages, warranting further validation in prospective, multicenter studies.

## Graphical abstract

Tocilizumab combined with high‐dose methylprednisolone improved outcomes in children with acute necrotizing encephalopathy. A dose of 30 mg/(kg·day) may be more effective than 20 mg/(kg·day).

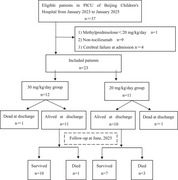

## INTRODUCTION

Acute necrotizing encephalopathy (ANE) is a devastating parainfectious encephalopathy that is frequently associated with viral infections such as influenza and coronavirus disease 2019. The mortality rates among children range from 29.4% to 66.7%, with approximately 0–30% of patients surviving without neurological disabilities.^1^, ^2^, ^3^ Although the etiology and pathogenesis of ANE remain unclear, a dysregulated immune response—commonly referred to as a cytokine storm—is widely considered the central mechanism.^2^, ^3^

Tocilizumab is a humanized anti‐human IL‐6 receptor monoclonal antibody that has demonstrated efficacy in modulating hyperinflammatory states across various immune‐mediated diseases.^4^, ^5^ Recent clinical studies suggest that early administration of tocilizumab combined with corticosteroids and intravenous immunoglobulin (IVIG) may improve outcomes in severe ANE cases.^6^, ^7^, ^8^ However, the optimal dosing strategy for methylprednisolone (MP) remains controversial, with clinical practice varying between 10 and 30 mg/(kg·day). Our multicenter retrospective analysis revealed that MP doses of ≥20 mg/(kg·day) are associated with reduced in‐hospital and post‐discharge mortality rates compared to lower doses.^9^ Despite this, no studies have directly compared the clinical efficacy and safety of 20 mg/(kg·day) versus 30 mg/(kg·day) regimens.

This study aimed to address this gap by evaluating the comparative effectiveness and safety of two high‐dose MP regimens [20 mg/(kg·day) vs. 30 mg/(kg·day)], both in combination with tocilizumab, in children with ANE. Findings from this study may inform standardized immunotherapy protocols for this life‐threatening condition.

## METHODS

### Ethical approval

The study protocol was reviewed and approved by the Institutional Review Board of Beijing Children's Hospital, Capital Medical University (approval number: 2019‐k‐198). The guardians of all children with ANE provided written consent for the administration of high‐dose steroid therapy.

### Patient population

This retrospective study was conducted in the pediatric intensive care unit (PICU) of Beijing Children's Hospital between January 2023 and January 2025. The inclusion criteria for the study cohort were as follows: 1) age between 29 days and 18 years; 2) diagnosis of ANE based on the neuroradiological findings according to the criteria proposed by Mizuguchi et al.^2^; and 3) initiation of combination therapy with high‐dose MP pulse [initial dose of either 20 mg/(kg·day) or 30 mg/(kg·day)] and tocilizumab. To allow adequate time for treatment initiation, clinical observation, and reliable data collection, patients who remained in the PICU for less than 24 h were excluded.

### Study design

Based on the initial MP dose, patients were stratified into a 20 mg/(kg·day) group and a 30 mg/(kg·day) group for comparative analysis of baseline characteristics, inflammatory markers, survival rates, and neurological outcomes.

### Data collection

Data on demographics, clinical manifestations, laboratory examination results, therapeutic schedules, adverse reactions, and clinical outcomes were collected. The demographic information included age, sex, family history, and underlying diseases. Clinical manifestations included symptoms, Glasgow Coma Scale (GCS), and ANE severity score (ANE‐SS)^10^ on admission.

Laboratory examination results, including C‐reactive protein (CRP), procalcitonin (PCT), serum and cerebrospinal fluid (CSF) interleukin‐1 beta (IL‐1β), IL‐2, IL‐4, IL‐6, IL‐8, IL‐10, tumor necrosis factor alpha (TNF‐α), routine blood test results, liver enzyme levels, coagulation parameters and routine CSF markers, were collected when patients were admitted to the PICU and 3 days after treatment. Cranial imaging and etiological tests (respiratory virus antigen or nucleic acid detection) were also performed.

### Treatment

Therapeutic schedules included conventional treatments (temperature control, mannitol, anticonvulsants, antivirals), organ support (mechanical ventilation, vasoactive drugs), and immunomodulation (MP, IVIG, tocilizumab, plasma exchange [PLEX], mild hypothermia). MP pulse therapy was administered for 3 days, followed by tapering over 7–10 days. IVIG was administered at 1 g/(kg·day) for 2 days. Tocilizumab was given intravenously at a standard dose based on body weight (≤30 kg: 12 mg/kg; >30 kg: 8 mg/kg), infused over ≥1 h. A second dose could be administered one week after the first if there was evidence of ongoing cytokine storm (e.g., persistent fever, elevated CRP or PCT, or clinical worsening). Heparin was used for catheter patency or when clinically indicated in patients with coagulation dysfunction. Adverse reactions were monitored during infusion.

### Safety assessments

Safety assessments focused on the monitoring of treatment‐related adverse effects. These included hormone‐related effects such as hyperglycemia, hypertension, thrombosis, and secondary infections; and tocilizumab‐related risks such as neutropenia, thrombocytopenia, rash, shock, liver dysfunction, allergic reactions, and gastrointestinal perforations.

### Follow‐up

Follow‐up was conducted via outpatient visits or telephone interviews to assess neurological function, rated using the Pediatric Overall Performance Category (POPC) scale^11^: 1 = complete recovery, 2 = mild impairment, 3 = moderate impairment, 4 = severe impairment, 5 = coma/vegetative state, and 6 = death. A POPC score of ≥4 indicates severe neurological sequelae.

### Statistical analysis

Continuous variables are expressed as the mean ± standard deviation or as the median (interquartile range), based on their parametric or nonparametric distribution. Categorical variables are expressed as a number or percentage. Student's *t*‐test was used for parametric continuous variables, and the Mann–Whitney *U* test was used for nonparametric continuous variables. Categorical variables were analyzed using the chi‐square test or Fisher's exact test. Statistical significance was set at *P* <0.05. All the statistical analyses were conducted using IBM SPSS software, version 27 (IBM Corp., Armonk, NY, USA).

## RESULTS

A total of 23 patients were included: 11 received 20 mg/(kg·day) MP and 12 received 30 mg/(kg·day) MP. Baseline characteristics were comparable between the groups (Table 1). The median time from prodromal symptoms to encephalopathy was 36.0 h (interquartile range: 19.5–48.0 h). Genetic mutations were found in five cases (21.7%), including four with *RANBP2* mutations and one with *CSMD1* mutations. One case (4.3%) had a history of developmental delay.

**TABLE 1:** Comparison of clinical characteristics between the two groups of children with acute necrotizing encephalopathy

Characteristics	Total patients (*n* = 23)	20 mg/(kg·day) (*n* = 11)	30 mg/(kg·day) (*n* = 12)	*P*‐value
Basic information				
Age (years)	5.0 (1.6, 8.1)	5.0 (1.0, 6.0)	4.0 (1.7, 9.5)	0.459
Gender (male)	8 (34.8)	2 (18.2)	6 (50.0)	0.193
Season (winter or spring)	19 (82.6)	9 (81.8)	10 (83.3)	1.000
Underlying disease	1 (4.3)	0 (0)	1 (8.3)	1.000
Pathogen				
Influenza A virus	16 (69.6)	7 (63.6)	9 (75.0)	0.667
Influenza B virus	1 (4.3)	0 (0)	1 (8.3)	1.000
SARS‐CoV‐2	3 (13.0)	1 (9.1)	2 (16.7)	1.000
Mycoplasma	2 (8.7)	2 (18.2)	0 (0)	0.217
HHV‐6	1 (4.3)	1 (9.1)	0 (0)	1.000
Severity scores on admission				
ANE‐SS	5.0 (5.0, 6.0)	5.0 (5.0, 6.0)	5.5 (4.3, 6.8)	0.752
GCS (≤5)	13 (56.5)	6 (54.5)	7 (58.3)	1.000
Clinical features				
Prodromal stage (hours)	36.0 (19.5, 48.0)	37.0 (20.0, 72.0)	27.3 (12.0, 45.0)	0.155
Temperature (°C)	40.0 (39.5, 41.0)	40.0 (39.5, 41.0)	39.9 (39.4, 41.0)	0.926
Disturbance of consciousness	23 (100.0)	11 (100.0)	12 (100.0)	1.000
Convulsion	19 (82.6)	8 (72.7)	11 (91.7)	0.317
Shock	6 (26.1)	3 (27.3)	3 (25.0)	1.000
MODS	9 (39.1)	4 (36.4)	5 (41.7)	1.000
Coagulation dysfunction	18 (78.3)	8 (72.7)	10 (83.3)	0.640
Brainstem	21 (91.3)	10 (90.9)	11 (91.7)	1.000

Data are presented as *n* (%) or median (interquartile range).

Abbreviations: ANE‐SS, acute necrotizing encephalopathy severity score; GCS, Glasgow Coma Scale; HHV‐6, human herpes virus 6; MODS, multiple organ dysfunction syndrome; SARS‐CoV‐2, severe acute respiratory syndrome coronavirus‐2.

At admission, 78.3% (18/23) were critically ill (ANE‐SS ≥ 5), 56.5% (13/23) were in deep coma (GCS ≤ 5), and 91.3% (21/23) had multiple organ dysfunction syndrome, including shock (65.2%, 15/23), coagulation disorders (65.2%, 15/23), and brainstem involvement (91.3%, 21/23). There were no significant differences in organ dysfunction or illness severity between groups (*P* > 0.05).

Respiratory pathogens were identified in all patients, with influenza A virus being the most prevalent (69.6%, 16/23), followed by severe acute respiratory syndrome coronavirus‐2 (13.0%, 3/23), *Mycoplasma pneumoniae* (8.7%, 2/23), human herpes virus 6 (4.3%, 1/23), and influenza B virus (4.3%, 1/23) (Table 1).

Peripheral blood and CSF tests were performed within 12 h of PICU admission for all patients. The results revealed markedly elevated levels of serum PCT, CSF protein, and CSF cytokines (IL‐6 and IL‐8). In contrast, serum CRP and cytokines (IL‐6, IL‐8, and IL‐10) were only mildly elevated. No significant differences were found between the two groups in inflammatory markers, liver enzyme levels, or coagulation parameters (*P* > 0.05) (Table 2).

**TABLE 2:** Laboratory findings of children with acute necrotizing encephalopathy upon admission to the two study groups

Variables	All patients (*n* = 23)	20 mg/(kg·day) (*n* = 11)	30 mg/(kg·day) (*n* = 12)	*P*‐value
Serum				
WBC(×10^9^/L)	4.9 (3.4, 7.5)	5.2 (3.1, 8.6)	4.4 (3.4, 6.7)	0.580
PLT(×10^9^/L)	124.0 (83.0, 184.0)	113.0 (73.0, 179.0)	134.5 (97.3, 188.5)	0.441
CRP (mg/L)	14.0 (10.0, 26.5)	19.0 (10.0, 35.5)	14.0 (10.0, 25.3)	0.557
PCT (ng/mL)	24.1 (0.6, 50.0)	38.1 (0.6, 50.0)	18.5 (3.5, 30.0)	0.423
Ferritin (ng/mL)	207.1 (110.8, 600.2)	192.2 (124.9, 10675.0)	222.0 (91.8, 563.4)	0.560
ALT (U/L)	77.6 (27.0, 267.4)	61.4 (22.7, 267.4)	102.9 (25.2, 258.9)	0.854
AST (U/L)	162.4 (51.5, 488.2)	75.5 (52.8, 488.2)	173.1 (49.9, 502.7)	0.806
LDH (U/L)	355.0 (275.0, 892.0)	583.0 (323.0, 892.0)	318.5 (266.3, 865.0)	0.538
Creatinine (mmol/L)	37.8 (21.3, 55.0)	38.7 (31.3, 57.0)	32.1 (18.9, 54.3)	0.450
APTT (s)	32.2 (21.3, 41.9)	32.1 (22.0, 48.0)	32.5 (19.2, 37.2)	0.870
PT (s)	14.9 (13.4, 18.2)	14.7 (13.4, 18.7)	16.9 (13.4, 18.2)	0.948
INR	1.3 (1.1, 1.6)	1.3 (1.1, 1.7)	1.5 (1.2, 1.6)	0.888
D‐dimer (ng/mL)	1.2 (0.6, 6.2)	1.7 (0.7, 7.6)	0.6 (0.4, 5.6)	0.375
IL‐1β (pg/mL)	2.4 (1.7, 6.7)	2.8 (2.4, 52.8)	2.4 (1.4, 5.5)	0.245
IL‐2 (pg/mL)	2.4 (1.8, 2.5)	2.4 (1.8, 2.5)	2.4 (1.7, 2.9)	0.653
IL‐4 (pg/mL)	1.5 (0.7, 2.4)	2.4 (1.1, 2.5)	1.0 (0.5, 2.4)	0.060
IL‐6 (pg/mL)	72.3 (10.6, 133.1)	73.5 (27.8, 523.3)	29.3 (6.9, 125.6)	0.233
IL‐8 (pg/mL)	46.6 (14.9, 169.6)	34.5 (14.9, 1613.9)	52.6 (18.9, 94.9)	1.000
IL‐10 (pg/mL)	20.7 (6.9, 87.6)	25.8 (5.0, 179.1)	20.7 (6.9, 33.5)	0.418
TNF‐α (pg/mL)	2.4 (1.8, 3.1)	2.4 (2.4, 3.1)	2.4 (1.6, 3.7)	0.622
IFN‐γ (pg/mL)	2.5 (0.7, 10.3)	5.0 (2.3, 23.2)	1.0 (0.6, 6.8)	0.093
Cerebrospinal fluid				
WBC (cells/L)	2.0 (0, 2.0)	1.0 (0, 4.0)	2.0 (1.0, 2.0)	0.495
Protein (mg/L)	1439.0 (640.3, 2182.3)	1258.0 (400.0, 2339.0)	1682.0 (712.0, 2130.0)	0.622
LDH (U/L)	37.0 (22.0, 56.8)	30.5 (17.3, 44.8)	45.5 (24.0, 66.3)	0.226
Sugar (mmol/L)	5.1 (4.1, 6.0)	5.7 (4.5, 6.7)	4.8 (4.0, 5.7)	0.239
Lactic acid (mmol/L)	1.7 (1.5, 2.5)	2.5 (1.6, 3.7)	1.6 (1.4, 2.1)	0.020
IL‐1β (pg/mL)	1.3 (0.7, 2.3)	2.1 (1.3, 3.5)	0.7 (0.7, 1.2)	0.010
IL‐2 (pg/mL)	1.8 (0.9, 3.2)	1.9 (0.1, 3.6)	1.7 (0.9, 2.6)	1.000
IL‐4 (pg/mL)	0.6 (0.4, 1.3)	0.7 (0.3, 1.6)	0.6 (0.4, 1.1)	0.958
IL‐6 (pg/mL)	645.7 (195.8, 950.5)	722.1 (111.0, 1568.1)	632.0 (203.8, 778.1)	0.453
IL‐8 (pg/mL)	1010.0 (493.1, 7128.0)	896.7 (21.1, 7500.0)	1066.0 (493.1, 7128.0)	1.000
IL‐10 (pg/mL)	14.7 (5.7, 28.2)	17.3 (5.4, 30.7)	6.9 (4.2, 15.0)	0.791
TNF‐α (pg/mL)	0.5 (0.2, 1.0)	0.3 (0.2, 0.5)	0.9 (0.6, 3.0)	0.010
IFN‐γ (pg/mL)	0.2 (0.1, 1.0)	0.5 (0.3, 1.3)	0.1 (0.1, 0.2)	0.034

Data are presented as *n* (%) or median (interquartile range).

Abbreviations: ALT, alanine aminotransferase; APTT, activated partial thromboplastin time; AST, aspartate aminotransaminase; CRP, C‐reactive protein; IFN‐γ, Interferon‐gamma; IL‐1β, interleukin‐1 beta; IL‐2, interleukin‐2; IL‐4, interleukin‐4; IL‐6, interleukin‐6; IL‐8, interleukin‐8; IL‐10, interleukin‐10; INR, international normalized ratio; LDH, lactate dehydrogenase; PCT, procalcitonin; PLT, platelet; PT, prothrombin time; TNF‐α, tumor necrosis factor alpha; WBC, white blood cell.

Pre‐ and post‐treatment inflammatory markers in both groups are presented in Table 3. In the 30 mg/(kg·day) group, PCT levels decreased significantly (18.5 vs. 0.1 ng/mL, *P* = 0.005), as did CSF protein (1682.0 vs. 441.5 mg/L, *P* = 0.025) and CSF cytokines: IL‐6 (632.0 vs. 81.5 pg/mL, *P* = 0.028), IL‐8 (1010.0 vs. 106.0 pg/mL, *P* = 0.028), and IL‐10 (14.3 vs. 0.8 pg/mL, *P* = 0.043). Comparable reductions were observed in the 20 mg/(kg·day) group, with significant decreases in PCT (38.1 vs. 0.2 ng/mL, *P* = 0.005), CSF protein (1258.0 vs. 488.5 mg/L, *P* = 0.050), and CSF cytokines: IL‐6 (722.1 vs. 38.7 pg/mL, *P* = 0.028), IL‐8 (896.7 vs. 102.9 pg/mL, *P* = 0.046), and IL‐10 (17.3 vs. 0.8 pg/mL, *P* = 0.028). Serum cytokine levels (IL‐6, IL‐8, and IL‐10) showed no statistically significant changes in either group (*P* > 0.05), despite a general downward trend.

**TABLE 3:** Differences in inflammatory indicators between the two study groups at admission and 3 days post‐treatment

	20 mg/(kg·day) (*n* = 11)			30 mg/(kg·day) (*n* = 12)		
Variables	Day 1	Day 3	*P*‐value	Day 1	Day 3	*P*‐value
Serum						
PCT (ng/mL)	38.1 (0.6, 50.0)	0.2 (0.1, 0.3)	0.005	18.5 (3.5, 30.0)	0.1 (0.1, 0.2)	0.005
IL‐6 (pg/mL)	73.5 (27.8, 523.3)	66.0 (7.5, 282.9)	0.779	29.3 (6.9, 125.6)	61.7 (13.3, 75.1)	0.893
IL‐8 (pg/mL)	34.5 (14.9, 1613.9)	18.8 (9.6,146.8)	0.237	52.6 (18.9, 94.9)	24.2 (7.0, 116.4)	0.144
IL‐10 (pg/mL)	25.8 (5.0, 179.1)	2.5 (2.4, 4.7)	0.128	20.7 (6.9, 33.5)	2.5 (2.3, 3.8)	0.068
Cerebrospinal fluid						
Protein (mg/L)	1258.0 (400.0, 2339.0)	488.5 (188.5, 599.3)	0.050	1682.0 (712.0, 2130.0)	441.5 (365.5, 570.0)	0.025
IL‐6 (pg/mL)	722.1 (111.0, 1568.1)	38.7 (11.3, 263.8)	0.028	632.0 (203.8, 778.1)	81.5 (32.8, 109.1)	0.028
IL‐8 (pg/mL)	896.7 (21.1, 7500.0)	102.9 (80.9, 260.6)	0.046	1010.0 (530.3, 4494.0)	106.0 (58.6, 308.1)	0.028
IL‐10 (pg/mL)	17.3 (5.6, 29.4)	0.8 (0.6, 1.7)	0.028	14.3 (8.2, 38.1)	0.8 (0.4, 2.7)	0.043

Data are presented as median (interquartile range).

Abbreviations: IL‐6, interleukin‐6; IL‐8, interleukin‐8; IL‐10, interleukin‐10; PCT, procalcitonin.

All patients started immunotherapy within 24 h of PICU admission. Of these, 95.7% (22/23) received MP treatment within 72 h of encephalopathy onset. All patients received the first dose of tocilizumab within 48 h after PICU admission, with 34.8% (8/23) requiring a second dose one week later. Most patients (91.3%, 21/23) required mechanical ventilation. Although the median duration of mechanical ventilation was shorter in the 30 mg/(kg·day) group than that in the 20 mg/(kg·day) group (162.0 vs. 250.5 h), the difference was not significant (*P* = 0.542). The two groups showed similar utilization rates of adjunctive therapies, including antivirals (91.3%, 21/23), IVIG (100%, 23/23), PLEX (43.5%, 10/23), and therapeutic hypothermia (47.8%, 11/23) (Table 4).

**TABLE 4:** Treatments and outcomes in children with acute necrotizing encephalopathy in the two study groups

Variables	20 mg/(kg·day) (*n* = 11)	30 mg/(kg·day) (*n* = 12)	*P*‐value
First steroid therapy after encephalopathy (hours)			
<24	7 (63.6)	8 (66.7)	1.000
<48	8 (72.7)	9 (75.0)	1.000
<72	10 (90.9)	12 (100.0)	0.478
Duration of steroid therapy (days)	3 (27.3)	3 (25.0)	1.000
Initiation time of tocilizumab after admission (hours)			
<24	8 (72.7)	9 (75.0)	1.000
<48	3 (27.3)	3 (25.0)	
Number of tocilizumab (times)			
1	6 (54.5)	9 (75.0)	0.400
2	5 (45.5)	3 (25.0)	
Combination therapy			
IVIG	11 (100.0)	12 (100.0)	1.000
PLEX	6 (54.5)	4 (33.3)	0.414
Mild hypothermia	6 (54.5)	5 (41.7)	0.684
Antiviral therapy	9 (81.8)	12 (100.0)	0.217
Mechanical ventilation	10 (90.9)	11 (91.7)	1.000
Duration of mechanical ventilation (hours)	250.5 (182.5, 316.0)	162.0 (144.0, 418.0)	0.526
Duration of PICU stay (days)	14.0 (9.0, 26.0)	10.0 (5.9, 27.3)	0.758
Length of stay (days)	20.0 (11.0, 32.0)	17.5 (10.5, 34.0)	0.926
Died at discharge	1 (9.1)	1 (8.3)	1.000
Died at the last follow‐up	4 (36.4)	2 (16.7)	0.371

Data are presented as *n* (%) or median (interquartile range).

Abbreviations: IVIG, intravenous immunoglobulin; PICU, pediatric intensive care unit; PLEX, plasma exchange.

Two patients (8.7%, 2/23) died during hospitalization. Cumulative mortality was lower in the 30 mg/(kg·day) group (16.7%) than in the 20 mg/(kg·day) group (36.4%), although the difference was not statistically significant (*P* = 0.371). Median PICU (14.0 vs. 10.0 days) and total hospital stays (20.0 vs. 17.5 days) were longer in the lower‐dose group, but these differences also lacked statistical significance (*P* > 0.05) (Table 4).

At 6–12 months of follow‐up, 5.9% of the surviving patients achieved full recovery, while 11.8%, 29.4%, and 52.9% had mild, moderate, and severe neurological sequelae, respectively. Four patients remained comatose. The incidence of severe neurological sequelae at discharge was significantly lower in the 30 mg/(kg·day) group than in the 20 mg/(kg·day) group (41.7% vs. 90.9%, *P* = 0.027) (Table 5).

**TABLE 5:** Pediatric overall performance category (POPC) scores at hospital discharge and during 6‐ to 12‐month post‐discharge follow‐up in the two study groups

POPC scores	20 mg/(kg·day) (*n* = 11)	30 mg/(kg·day) (*n* = 12)	*P*‐value
At discharge (*n* = 23)			
1–2	0 (0)	1 (8.3)	1.000
3	1 (9.1)	6 (50.0)	0.069
4–6	10 (90.9)	5 (41.7)	0.027
Follow up at 6–12 months (*n* = 17)			
1–2	0 (0)	3 (30.0)	0.228
3	1 (14.3)	4 (40.0)	0.338
4–6	6 (85.7)	3 (30.0)	0.050

Data are presented as *n* (%).

Secondary infections occurred in 47.8% (11/23) of patients, including fungal (*n* = 2), bacterial (*n* = 5), and mixed infections (*n* = 4). Thrombotic complications were reported in 21.7% (5/23) of patients, including cerebral artery embolism (*n* = 2) and limb venous thrombosis (*n* = 3). Reactive hyperglycemia occurred in 13.0% (3/23) of patients during MP pulse therapy. No statistically significant difference in adverse events was found between the groups (*P* = 1.000) (Table 6). No tocilizumab‐related adverse events were identified.

**TABLE 6:** Possible adverse reactions in children with acute necrotizing encephalopathy across the two study groups

Adverse reactions	20 mg/(kg·day) (*n* = 11)	30 mg/(kg·day) (*n* = 12)
Secondary infection	5 (45.5)	6 (50.0)
Bacterial	2 (18.2)	4 (33.3)
Fungal	3 (27.3)	4 (33.3)
Thrombosis	2 (18.2)	3 (25.0)
Hyperglycemia	1 (9.1)	2 (16.7)
Hypertension	0 (0)	1 (8.3)

Data are presented as *n* (%).

## DISCUSSION

ANE is a severe pediatric critical neurological illness triggered by a cytokine storm, with early combined immunomodulatory therapy serving as its first‐line treatment.^12^, ^13^ Recent clinical studies have shown that the combination of tocilizumab, high‐dose glucocorticoids, and IVIG improves the prognosis of children with ANE.^6^, ^7^, ^8^ However, the efficacy and safety of this approach may vary with the glucocorticoid dosage. This study evaluated the use of tocilizumab as an adjuvant therapy in conjunction with two high‐dose MP pulse regimens [20 mg/(kg·day) vs. 30 mg/(kg·day)]. Compared with the 20 mg/(kg·day) regimen, the 30 mg/(kg·day) MP regimen demonstrated comparable effectiveness in terms of mortality, anti‐inflammatory response, duration of ventilation, length of hospital stay, and infection rates, while exhibiting a lower incidence of severe neurological sequelae.

The reported ANE mortality rate ranges from 30% to 40%, reaching 60%–70% in severe cases.^1^, ^2^, ^3^, ^8^ In addition to disease severity, the choice of immunotherapy significantly impacts the clinical outcomes of ANE. Tocilizumab, an IL‐6 receptor antagonist, has shown promise in managing disorders associated with cytokine storm. A Chinese multicenter study revealed that five of eight critically ill ANE children treated with tocilizumab, MP [10–20 mg/(kg·day)], and IVIG within 24 h of PICU admission survived.^8^ Similarly, a Malaysian retrospective study found that all seven critically ill ANE patients treated with tocilizumab and 30 mg/(kg·day) MP survived.^6^ In our study, 78.3% (18/23) of patients were high‐risk ANE (ANE‐SS ≥ 5), and the overall mortality rate was 26.1%. This rate is lower than that reported in most historical studies, supporting the efficacy of early tocilizumab combined with high‐dose steroid therapy. However, the specific impact of steroid dosage on outcomes remains unclear due to a lack of direct comparisons. Prior studies report ANE mortality rates ranging from 17% to 48.5% with an initial MP dose of 20 mg/(kg·day) plus IVIG,^9^, ^14^ compared to rates of 25% to 42.3% with 30 mg/(kg·day).^1^, ^15^, ^16^, ^17^ In our cohort, the 30 mg/(kg·day) group had a lower mortality rate than the 20 mg/(kg·day) group (16.7% vs. 36.4%). Although this difference was not statistically significant—likely due to the small sample size—the observed trend suggests a potential survival benefit with the higher MP dose and may provide valuable direction for future clinical studies.

The pathogenesis of ANE is driven by a cytokine storm resulting from dysregulated immune responses to infection. Elevated levels of TNF‐α, IL‐6, and IL‐8 correlate with disease severity and prognosis.^18^, ^19^ In line with this, our study found elevated levels of IL‐6, IL‐8, and IL‐10 in both the serum and CSF of ANE patients, with CSF IL‐6 and IL‐8 showing particularly marked increases. Tocilizumab reduces neuroinflammation by blocking IL‐6 signaling. Glucocorticoids suppress pro‐inflammatory cytokines via cytoplasmic receptor binding, although their precise mechanism in ANE treatment remains unclear. The combined immunotherapy regimen significantly reduced CSF protein and pro‐inflammatory cytokine (IL‐6 and IL‐8) levels. In contrast, serum IL‐6 and IL‐8 levels showed only a downward trend without reaching statistical significance, likely attributable to their initially mild elevation. Early serum PCT was elevated in both groups, consistent with the findings of Li et al.,^9^ suggesting a potential link to the cytokine storm or sepsis. PCT may serve as an early warning biomarker for ANE and correlates with prognosis.^12^, ^19^ In our study, pulse therapy with MP at doses of either 20 mg/(kg·day) or 30 mg/(kg·day) significantly decreased serum PCT levels within 3 days, demonstrating effective control of the inflammatory cascade.

Most survivors of ANE experience neurological sequelae, with over half having severe outcomes.^20^, ^21^ In our cohort, only 17.6% (3/17) of surviving patients had favorable outcomes (POPC score = 1–2), while 52.9% (9/17) showed severe deficits, including consciousness disorders, motor dysfunction, language delay, and refractory epilepsy. Zhu et al.^14^ reported that 76% of patients treated with 20 mg/(kg·day) steroids developed severe sequelae, with no cases of complete recovery. Lee et al.^20^ found that among patients receiving 30 mg/(kg·day) steroids, 42% had severe sequelae, 25% showed partial magnetic resonance imaging abnormalities with preserved function, and one case fully recovered. These results suggest that higher initial steroid doses may improve neurological prognosis in ANE. Consistent with this, the severe sequelae rate at discharge was significantly lower in our 30 mg/(kg·day) group than in the 20 mg/(kg·day) group. However, given the potential influence of rehabilitation duration, long‐term follow‐up is necessary to confirm these effects.

The observed reduction in severe neurological sequelae, without a concurrent significant difference in mortality, may reflect distinct pathophysiological processes in ANE. Early mortality is often driven by fulminant cerebral edema, brainstem herniation, or multi‐organ failure—conditions that may be irreversible once established. In contrast, long‐term neurological outcomes are closely linked to subacute inflammatory injury, demyelination, and axonal damage, processes that are potentially modifiable by timely and potent anti‐inflammatory intervention. High‐dose MP exerts stronger suppression of pro‐inflammatory cytokines, stabilizes the blood‐brain barrier, and reduces microglial activation, thereby limiting secondary neuronal damage.^22^, ^23^ Thus, while both regimens may fail to prevent early fatal events, the 30 mg/(kg·day) regimen may offer superior neuroprotection during the critical window of immune‐mediated injury.

Safety data for immunomodulatory therapy in ANE remain limited. High‐dose steroid pulse therapy may cause adverse effects, including infections, hypercoagulability, glucose metabolism disorders, and abnormal blood pressure. In this study, 11 patients developed secondary bacterial or fungal infections, and five had thrombosis (including two with cerebral artery embolism). Invasive procedures such as PLEX may increase hypercoagulability risk. Among three patients with limb venous thrombosis who did not receive PLEX, elevated D‐dimer levels were observed. Reactive hyperglycemia occurred in three patients during steroid treatment, while hypertension was uncommon, potentially attributable to intracranial pressure management. Tocilizumab may cause adverse events such as liver dysfunction, allergic reactions, or rare gastrointestinal perforation. In this cohort, real‐time monitoring during infusion revealed no acute reactions (e.g., chills, allergies, hypotension), and no severe tocilizumab‐related adverse events were documented during follow‐up.

This study has several limitations. The single‐center design and relatively small sample size may limit statistical power, and the retrospective nature could lead to incomplete data collection and potential confounding due to combined therapies. Nevertheless, given the rarity of ANE, this cohort study provides valuable insights into its clinical characteristics and outcomes. Future research should involve prospective, multicenter studies with standardized longitudinal data collection and stratified randomization based on disease severity or pathogen type to enhance comparability and control for confounding factors.

A combination of tocilizumab, high‐dose MP, and IVIG appears to be an effective and safe therapeutic approach for ANE. Notably, the 30 mg/(kg·day) MP regimen was associated with significantly better neurological outcomes compared to the 20 mg/(kg·day) dose, without an increase in adverse events, indicating a potential neuroprotective advantage at the higher dose. Prospective, large‐scale studies are needed to confirm these findings and help establish an optimal treatment protocol for ANE.

## CONFLICT OF INTEREST

The authors declare no conflict of interest.
